# Bilateral staghorn kidney stones in Megacalycosis: Non operative management of complex kidney stone disease

**DOI:** 10.1016/j.eucr.2022.102146

**Published:** 2022-06-26

**Authors:** Charles J. O’ Connor, Ned Kinnear, Gemma Browne, Derek B. Hennessey

**Affiliations:** a Mercy University Hospital, Cork, Ireland; b Department of Urology, St Vincent's Hospital Melbourne, Melbourne, Australia

**Keywords:** Megacalycosis, Management, Complex, Kidney, Stones, Calculi

## Abstract

What happens when kidney stone clearance is not feasible? We report the case of a 46-year-old male who presented for review with bilateral congenital non-obstructive calyceal dilatation (megacalycosis) and high volume bilateral renal calculi in the setting of stage four chronic kidney disease. Since complete stone clearance was deemed futile, thus a consensus was made between Urology and Nephrology, and treatment goals were focused on addressing symptoms, preserving renal function and preventing urinary tract infections until renal transplantation is needed. This case highlights that for some patients with severe complex kidney stone disease, an alternative management plan is needed.

## 1 Introduction

Megacalycosis or Puigvert Disease is the congenital non-obstructive enlargement of renal calyces in the presence of an unobstructed ureter and renal pelvis.^1^ The condition's hallmarks are hypoplastic renal papillae surrounding rather than imprinting the dilated calyces. Pathogenesis is poorly understood but may relate to dysgenesis of the ureteral bud and metanephros. Megacalycosis is usually diagnosed because of its complications including calculi or urinary tract infections (UTI).

## 2 Case presentation

**Figure 1:**
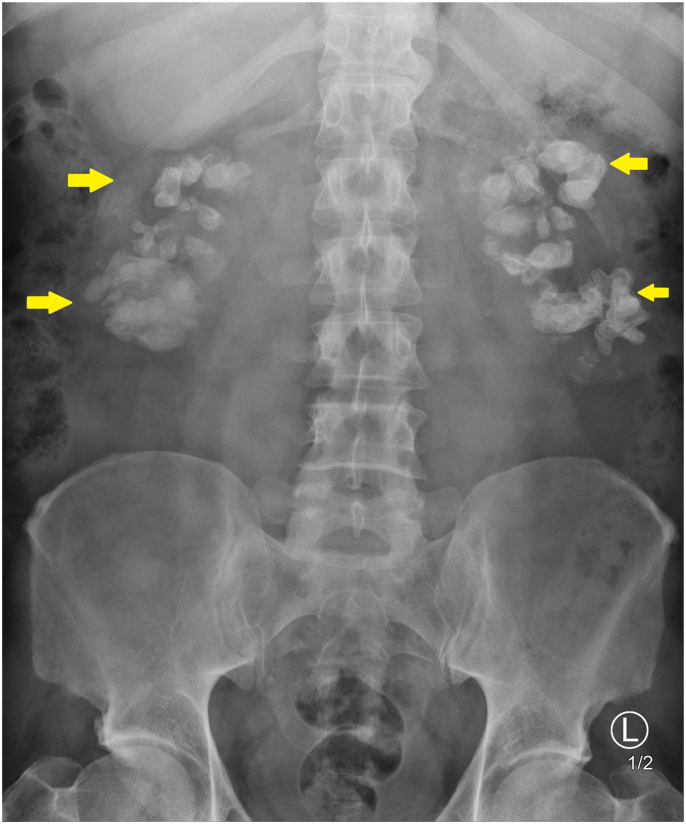
X-ray KUB of the abdomen. Significant bilateral calcifications are shown to be projected over each kidney.

**Fig. 2:**
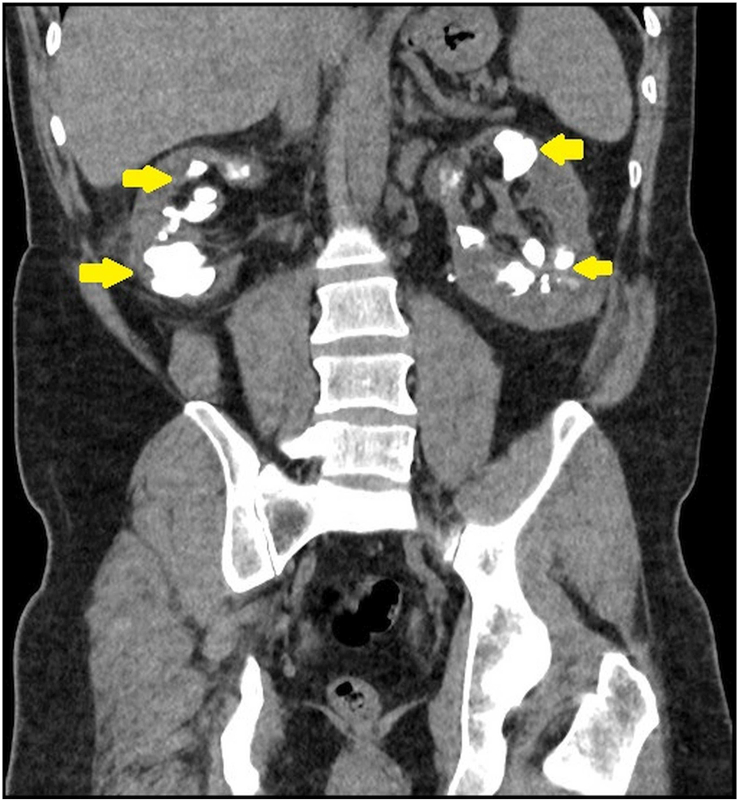
Non contrast computed tomography of the abdomen and pelvis, coronal plane. There are bilateral dilated renal calcyes, each filled with calculi. A semilunar shape of the calyces along with their high number compared to a normal kidney can be seen. Renal parenchyma appears to be conserved in contrast to classical staghorn calculi.

A 46-year-old male was referred from the nephrology service for an opinion regarding severe bilateral renal stone disease. He reported lifelong recurrent bilateral flank pain and UTIs. He denied any visible haematuria or weight loss. His co-morbidities included stage four chronic kidney disease (CKD). He had a lifelong history of recurrent renal calculi with multiple previous procedures in different centres from the age of 17. Urine culture grew gram-negative bacilli, 60,000 organisms per ml. Serum investigations revealed creatinine 245 μmol/L (NR 62–115), Estimated glomerular filtration rate (eGFR) 24.8 ml/min/1.73 m2, corrected calcium Ca 2.23 mmol/L (NR 2.12–2.63), uric acid 440 μmol/l (NR 202–416), parathyroid hormone (PTH) 109 μmol/L (NR 10–55). This patient's 24-h urine collection was within normal limits. An x-ray Kidney ureter and bladder (KUB) was available, and an up-to-date CT KUB was ordered. These images are shown in Fig. 1. & Fig. 2.

There are several etiologies that could cause this degree of stone disease. Urinary culture grew gram-negative bacilli. These could be urease splitting organisms, and the kidney stones could be struvite. Secondly, the serum PTH was mildly elevated; however, the PTH level was associated with normal serum calcium. It was felt that the elevated PTH was related to renal hyperparathyroidism. Uric acid levels were elevated; however, the stones were radiopaque on x-ray KUB. Medullary Sponge Kidney (MSK) is also a possible differential but was excluded on the radiological appearance. Instead, the CT KUB showed bilateral dilated multiple >30 calyces in each kidney. Each was filled with calculi. A diagnosis of megacalycosis with severe renal stone disease was made.

The case was discussed at a Urolithiasis multi-disciplinary team (MDT) meeting. It was felt that major surgery like anatrophic nephrolithotomy or percutaneous nephrolithotomy had the best chance of stone clearance. However, these were discounted due to the risk of these operations worsening the patient's CKD or requiring the patient to have a blood transfusion. It was felt that renal transplantation was ultimately needed, and the development of antibodies due to transfusion could make that more complicated. By consensus, it was decided that the treatment goals should be focused on delaying the need for renal transplantation for as long as possible. Efforts would be directed to maintain renal function, prevent UTIs and manage symptoms.

**Fig. 3:**
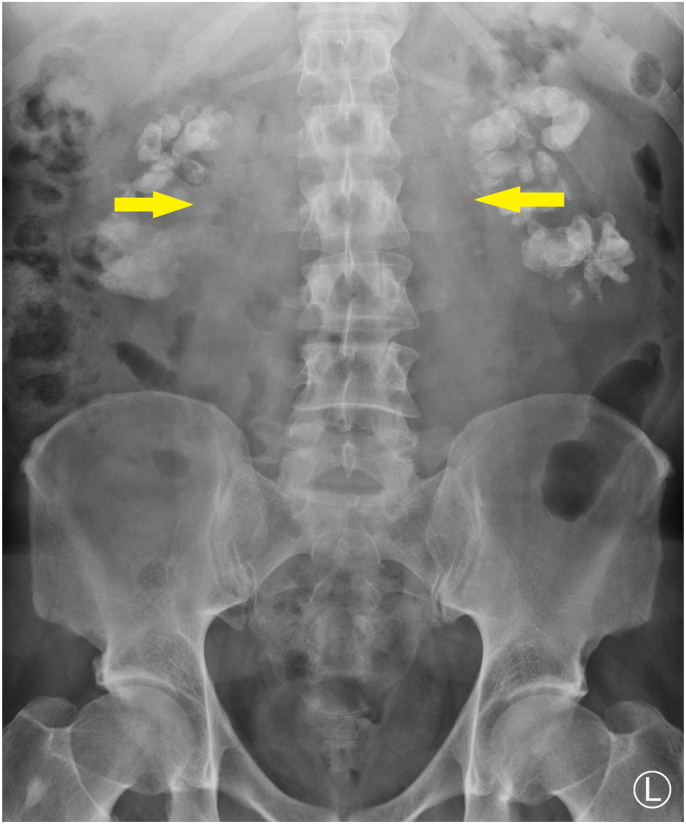
Post-operative plain film of the abdomen. Decreased stone burden in each renal pelvis is demonstrated.

Previous experience with this condition showed that clearance of the stone burden from the renal pelvis is beneficial. To do this, staged bilateral flexible ureteroscopy and laser lithotripsy were performed. Operative time for both ureteroscopies was between 50 and 60minutes utilizing a 273μm 30 W Holmium:YAG laser. Post-operative imaging of the abdomen demonstrated a reduction in renal calcification (Fig. 3). Creatinine improved to 217 μmol/L and the eGFR to 32 ml/min/1.73 m2. The stone analysis showed struvite 60%, carbapatite 15%, carbonated amorphous calcium phosphate 10%, calcium oxalate monohydrate 5%, and triglycerides 10%. The patient will have a regular outpatient follow-up with monitoring or renal function and imaging. Again, repeat flexible ureteroscopy, and laser lithotripsy will be performed whenever there is renal pelvic stone. The patient and a family member have also been referred to renal transplant services for workup for living donor transplantation.

## 3 Discussion

Megacalycosis is an uncommon congenital anomaly of renal development characterised by calyceal dilatation, increased calyces, and a normal renal pelvis. It is thought to occur due to the dysgenesis of renal pyramids, including a possible calyceal tissue weakness due to high pressure in the renal pelvis and a loss of the peristaltic function of the renal calyces. The megacalycose renal pelvis is usually normal with no smooth muscle hypertrophy. Histopathological analysis of an affected kidney will show a standard thickness of the renal cortex and sparse medulla; this would differentiate it from hydronephrotic tissue, where degeneration would be seen owing to elevated intrarenal pressure.^2^ The condition shows a male predominance of 6:1. Megacalycosis is associated with the Schinzel-Giedion syndrome, a rare condition in which midfacial retraction and skeletal abnormalities exist.

Megacalycosis can be unilateral or bilateral. All cases described by Puigvert were unilateral, while a case series of 30 patients showed 24 of the patients to have bilateral megacalycosis. Renal calculi were present in 14 of these 30 patients.^1^^,^^3^ In a separate case series of 11 patients, only one patient had bilateral megacalycosis and renal calculi.^4^ This review of the literature highlights the unique nature of our patient's case in which there are numerous bilateral kidney stones in the setting of renal impairment. Chronic kidney disease is not commonly reported with this condition, and the renal impairment in our case is most likely due to significant stone burden, to which the extent has not previously been reported in the literature.

Noteworthily-no official guidelines exist in managing this condition. The condition's treatment involves controlling symptoms in the form of urinary tract infections and recurrent nephrolithiasis. Radical treatment options like nephrectomy or pyeloplasty are usually contraindicated as many patients will have normally functioning kidneys.

Instead, flexible ureteroscopy and laser lithotripsy were chosen as the preferred method as it is a minor intervention in this case. Only stone in the renal pelvises was treated, as a stone extension into the renal pelvis was thought to be the culprit for obstruction. This intervention was straightforward and complication-free. Drainage improved, and eGFR improved. However, the prognosis is guarded in this case, and the need for renal transplantation is expected at some stage.

## 4 Conclusion

Megacalycosisis with severe bilateral stone disease and chronic kidney disease is a rare phenomenon. Traditional methods of kidney stone management had to be abandoned. Instead, the focus was to improve drainage, prevent UTI and delay the time until renal transplantation is needed.

## Authorship contribution

Charles O’ Connor: Conceptualization, Writing - Original Draft, Writing - Review & Editing, Ned Kinnear: Writing - Original Draft, Writing - Review & Editing, Gemma Browne: Conceptualization, Writing - Original Draft, Writing - Review & Editing, Derek Hennessey: Conceptualization, Writing - Original Draft, Writing - Review & Editing.

## Funding

This research did not receive any specific grant from funding agencies in the public, commercial, or not-for-profit sectors.

## Declaration of competing interest

None.
